# The Antiepileptic Drug Oxcarbazepine Inhibits the Growth of Patient-Derived Isocitrate Dehydrogenase Mutant Glioma Stem-like Cells

**DOI:** 10.3390/cells12081200

**Published:** 2023-04-20

**Authors:** Philip Dao Trong, Gerhard Jungwirth, Andreas Unterberg, Christel Herold-Mende, Rolf Warta

**Affiliations:** Division of Experimental Neurosurgery, Department of Neurosurgery, University Hospital Heidelberg, INF400, 69120 Heidelberg, Germany; philip.daotrong@med.uni-heidelberg.de (P.D.T.);

**Keywords:** isocitrate dehydrogenase mutation, antiepileptic drugs, repurposing drug screen, glioma stem cells, oxcarbazepine, perampanel

## Abstract

**Simple Summary:**

Patients diagnosed with isocitrate dehydrogenase (IDH)-mutated brain tumors frequently suffer from seizures, and the seizures were shown to promote tumor growth. Anti-seizure medications (antiepileptic drugs) might be able to break this vicious circle. However, it is not yet known which antiepileptic drugs might have additional anti-tumor effects. In this study, 20 commonly used antiepileptic drugs were tested on patient-derived tumor models. Only oxcarbazepine promoted additional tumor cell killing, making it an interesting drug to use in this special population of brain tumor patients.

**Abstract:**

Patients diagnosed with isocitrate dehydrogenase mutant (IDH^mut^) gliomas suffer frequently from seizures. Although the clinical course is less aggressive than that of its IDH wildtype counterpart, recent discoveries have shown that epileptic activity can promote tumor proliferation. However, it is not known if antiepileptic drugs confer additional value by inhibiting tumor growth. In this study, the antineoplastic properties of 20 FDA-approved antiepileptic drugs (AEDs) were tested in six patient-derived IDH^mut^ glioma stem-like cells (GSCs). Cell proliferation was assessed using the CellTiterGlo-3D assay. Two of the screened drugs (oxcarbazepine and perampanel) demonstrated an antiproliferative effect. A subsequent eight-point dose–response curve proved the dose-dependent growth inhibition for both drugs, but only oxcarbazepine reached an IC_50_ value below 100 µM in 5/6 GSCs (mean 44.7 µM; range 17.4–98.0 µM), approximating the possible c_max_ for oxcarbazepine in patient serums. Furthermore, the treated GSC spheroids were 82% smaller (mean volume 1.6 nL vs. 8.7 nL; *p* = 0.01 (live/dead^TM^ fluorescence staining)), and the apoptotic events increased by more than 50% (caspase-3/7 activity; *p* = 0.006). Taken together, this drug screen of a large series of antiepileptic drugs identified oxcarbazepine as a potent proapoptotic drug in IDH^mut^ GSCs, which combines antiepileptic and antineoplastic properties to treat this seizure-prone patient population.

## 1. Introduction

Patients diagnosed with isocitrate dehydrogenase mutant (IDH^mut^) gliomas suffer from disproportionally high rates of epileptic seizures, and often this symptom leads to the diagnosis of the brain tumor [1]. The recurrence or worsening of the epilepsy following first-line anti-tumor treatments often predicts the progression of the tumor [2]. This has a profound impact on the patients’ quality of life [3]. The patients are not allowed to drive and are, therefore, very limited in their mobility. Furthermore, tumor-associated epilepsy (TAE) increases the socioeconomic impact, as it often precludes a successful return to work and normal life, e.g., due to frequent relapses of seizures that eventually may become refractory to common antiepileptic medications. On the other hand, anti-tumor treatments often result in an improvement in epileptic seizure control. Surgical excision of the tumor represents one of the first treatment options [4,5]. Depending on the glioma entity, adjuvant chemoradiation can have further benefits in the treatment of TAE [6,7]. While the causality between tumor burden and epilepsy is undeniable, the effects of seizures on tumor proliferation are not well understood. Current studies suggest that glioma cells and neurons form an electric network that consists of synapses and gap junctions and, thereby, can foster cancer cell proliferation [8,9,10]. Through a positive feedback loop, tumor proliferation is promoted. Interrupting this feedback loop may mitigate tumor growth. Antiepileptic drugs might be the right candidate to interfere in this signaling cascade, especially because they are able to cross the blood–brain barrier. A drug that combines antiepileptic and antiproliferative properties would, therefore, be an optimal adjunct to existing chemotherapeutic regimens in the treatment of seizure-prone IDH^mut^ glioma patients.

Here, we used six well-characterized patient-derived IDH^mut^ glioma stem-like cells (GSCs) representing various aggressive glioma subtypes to evaluate the growth inhibition and apoptosis induced by 20 FDA-approved antiepileptic drugs [11,12,13]. Oxcarbazepine and perampanel demonstrated dose-dependent growth inhibition of IDH^mut^ GSCs, although only oxcarbazepine reached relevant IC_50_ levels.

## 2. Materials and Methods

### 2.1. Cell Culture and Antiepileptic Drug Selection

The patient-derived IDH^mut^ GSCs were cultivated as described before (Table 1) [14]. In brief, the GSCs were cultivated as spheroids in tumor stem cell (TSC) media that consisted of DMEM/F12 (Invitrogen, Karlsruhe, Germany), which contained 20% BIT serum-free supplement (Pelo-Biotech, Plannegg, Germany), 20 ng/mL basic fibroblast growth factor (bFGF), and 20 ng/mL epidermal growth factor (EGF) (Provitro, Berlin, Germany). The GSCs were authenticated by short tandem repeat (STR) profiling (DMSZ, Braunschweig, Germany). The characterization of the GSCs was described in previous studies [12,13,15]. D-2-hydroxyglutarate, an oncometabolite that is exclusively synthesized by the mutated IDH enzyme, was measured in previous experiments [13]. The AEDs were selected after literature research and clinical application and ordered from Sigma-Aldrich (St. Louis, MO, USA) and Selleckchem (Houston, TX, USA). The stock solutions were prepared according to the manufacturer’s instructions (gabapentin in H_2_O and the remaining AEDs in DMSO).

### 2.2. Cell Proliferation and Viability

The spheroids were mechanically and enzymatically (Accutase, Sigma-Aldrich) dissociated into a single-cell suspension and plated at 8000 cells per well in a 96-well plate in TSC medium. After 24 h, the AEDs were added to the GSCs at a concentration of 10 µM for 72 h. In the second step, the half-maximal inhibitory concentration values (IC_50_) of the selected drugs were determined. For this purpose, the experiments were performed in the following eight-step concentration range: 0.1 µM, 0.3 µM, 1 µM, 3 µM, 10 µM, 30 µM, 50 µM, and 100 µM. The cell proliferation was analyzed using the CellTiterGlo^®^-3D cell viability assay (Promega, Madison, WI, USA). The ready-to-use mix was added after 72 h of drug incubation, and the luminescence was measured on a Tecan Infinite 200 reader (Tecan, Männedorf, Switzerland) and normalized to the DMSO control. The experiments were performed in biological and technical triplicates.

### 2.3. Fluorescence Imaging

The induction of apoptosis was tested at the IC_30_ (30 µM) for NCH620, and fluorescent imaging was performed using a live/dead staining kit (Invitrogen). Equivalent to the viability assay, the spheroids were dissociated into a single-cell suspension and plated at 8000 cells per well in a 96-well plate in TSC medium. The drugs were added after 24 h. After 72 h, the spheroids were then incubated for 20 min with a two-component live/dead staining mix. The spheroids of each well were then photographed with an Olympus IX51 microscope, equipped with an XM10 camera (Olympus, Tokyo, Japan). The Olympus cellSens Dimension software (version 1.9) was used for the image acquisition.

### 2.4. Apoptosis Assay

The rate of apoptosis was measured in a time-dependent manner by assessing the caspase-3/7 activity at the IC_30_ (30 µM) with a luciferase-based caspase-3/7 Glo assay (Promega). The ready-to-use mastermix was added at different timepoints (12 h, 24 h, 36 h, and 48 h), and the luminescence signal was acquired using the Tecan Infinite 200 reader and normalized to the DMSO control within each timepoint.

### 2.5. Statistical Analysis

GraphPad Prism 7 (San Diego, CA, USA) was used to calculate the dose–response curves using normalized nonlinear regression with a sigmoid dose–response curve to obtain the IC_50_ values. A Student’s *t*-test was applied to test for differences in the spheroid volumes and induction of apoptosis. Significance was considered if *p* < 0.05.

## 3. Results

### 3.1. Antiproliferative Effect of AEDs in IDH^mut^ Glioma Stem-like Cells

**Figure 1:**
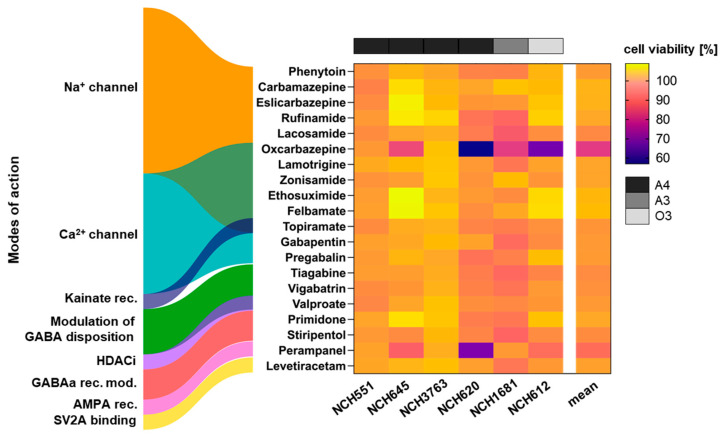
Drug screen for antineoplastic drug activity in IDH^mut^ GSCs representing aggressive IDH^mut^ gliomas. A total of 20 antiepileptic drugs (AEDs) were tested for antiproliferative properties at a concentration of 10 µM. Most AEDs are thought to exert their antiepileptic effect by modifying the electrolyte flux through the neuronal cell membrane, although overlapping modes of action exist, as depicted by the overlapping colors. Oxcarbazepine and perampanel show an antiproliferative effect in several GSCs, as depicted by the violet coloring in the heatmap. The experiments were performed in biological and technical triplicates. Rec. = receptor; mod. = modifier; GABA = gamma-aminobutyric acid; HDACi = histone deacetylase inhibitor; AMPA = α-amino-3-hydroxy-5-methyl-4-isoxazolpropionic acid; SV2A = synaptic vesicle glycoprotein 2A; A4 = astrocytoma WHO grade 4; A3 = astrocytoma WHO grade 3; O3 = oligodendroglioma WHO grade 3.

**Figure 2:**
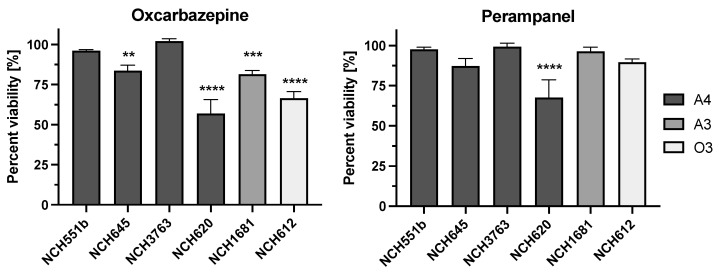
Antiproliferative effect of oxcarbazepine and perampanel at 10 µM. A significantly lower cell viability upon treatment with oxcarbazepine was observed in four out of the six GSCs compared to the DMSO control. Perampanel induced a significant decrease in viability only in one GSC (NCH620). The experiments were performed in biological and technical triplicates. A4 = astrocytoma WHO grade 4; A3 = astrocytoma WHO grade 3; O3 = oligodendroglioma WHO grade 3; ** *p* ≤ 0.05; *** *p* ≤ 0.001; **** *p* ≤ 0.0001.

To test the AEDs for antineoplastic activity, six well-characterized IDH^mut^ GSCs, which have been previously used in drug testing and are representative of various aggressive IDH^mut^ gliomas, were chosen (Table 1). Four of the analyzed GSCs (NCH551b, NCH645, NCH3763, and NCH620) were derived from patients suffering from recurrent astrocytoma WHO grade 4, and two cultures were derived from recurrent astrocytoma WHO grade 3 (NCH1681) and recurrent oligodendroglioma WHO grade 3 (NCH612) tumors. The cell viability was measured using the CellTiterGlo-3D assay at a concentration of 10 µM. Most of the tested AEDs did not induce a decrease in proliferation upon treatment of the GSCs, which is visualized by the mostly yellow/orange heatmap in Figure 1. However, oxcarbazepine demonstrated a significant reduction in cell viability in four out of the six GSCs compared to the DMSO control (NCH645 16.4% (*p* = 0.0028); NCH620 43% (*p* < 0.0001); NCH1681 18.5% (*p* = 0.0006); NCH612 33.5% (*p* < 0.0001); Figure 2). Furthermore, treatment with perampanel caused a significantly decreased cell viability only in NCH620, while the reduction observed for NCH645 did not reach statistical significance (NCH620 32.4%, *p* < 0.0001; NCH645 12.3%, *p* = 0.135; Figure 2).

### 3.2. Dose-Dependent Growth Inhibtion by Oxcarbazepine and Perampanel

**Figure 3:**
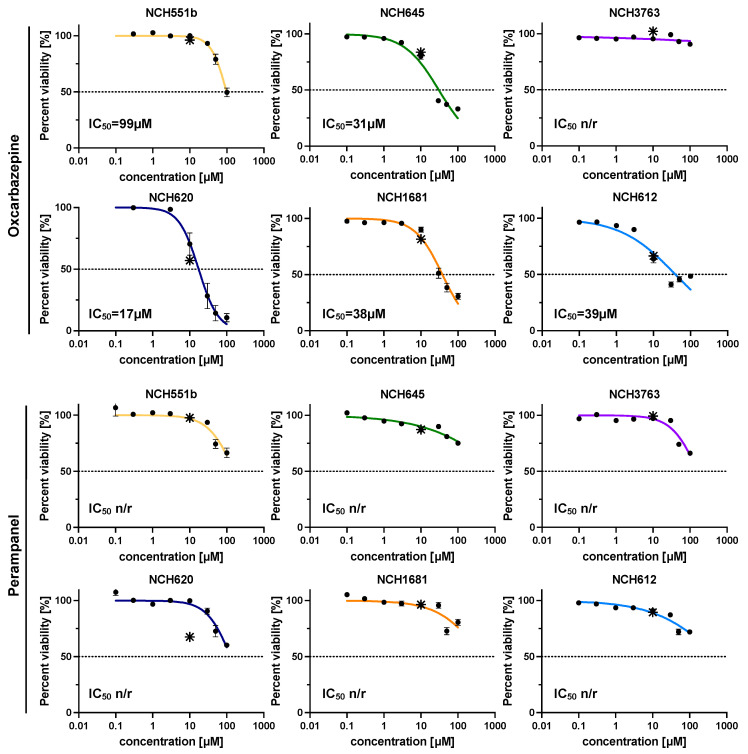
Dose–response curves for oxcarbazepine and perampanel in eight half-log concentration steps (0.1–100 µM). Both AEDs showed a dose-dependent growth inhibition, with oxcarbazepine reaching the IC_50_ in 5/6 IDH^mut^ GSCs and the lowest IC_50_ of 17.4 µM in NCH620. Perampanel induced a dose-dependent growth inhibition but did not reach the IC_50_ in the range of concentrations tested. The experiments were performed in biological and technical triplicates. * depicts the percent of cell viability in the initial drug screen (10 µM); n/r = not reached.

Next, we tested the dose-dependency of the anti-tumor response for oxcarbazepine and perampanel in an eight-step half-log concentration series with a range of 0.1–100 µM for all GSCs (Figure 3). Oxcarbazepine demonstrated a mean IC_50_ value of 44.7 µM in 5/6 GSCs (17.4–98.6 µM), with NCH620 being the most sensitive GSC (17.4 µM). Perampanel also showed a dose-dependent response but did not reach the IC_50_ value in the range of the concentrations tested. Nevertheless, the measured GSC sensitivities were in good agreement with the results of the initial screening (Figure 3; asterisks).

Since orally administered oxcarbazepine is regarded as a prodrug, the first-pass effect via the liver biotransforms it to its active metabolite, monohydroxyoxcarbazepine (MHD). However, because oxcarbazepine is a racemic mixture of two enantiomers, its activation results in two metabolites with an opposite three-dimensional shape that share the same connectivity, namely (R)-MHD and (S)-MHD (eslicarbazepine) [16]. Each enantiomer eventually encompasses different pharmacologic modes of action. Therefore, besides oxcarbazepine, the aforementioned metabolites might also be responsible for the observed growth inhibition of the IDH^mut^ GSCs. Eslicarbazepine, or (S)-MHD, is an FDA-approved AED and was, therefore, already included in our initial screen. Interestingly, it did not show an effect in any of the GSCs. To further elucidate this finding, we also tested the (R)-MHD metabolite in the GSCs, in which oxcarbazepine had shown an effect (Supplementary Figure S1). A significant inhibitory effect upon treatment with (R)-MHD could not be detected for concentrations up to 100 µM. Since the enzymes responsible for the metabolization of oxcarbazepine (AKR1C1, AKR1C2, AKR1C3, and AKR1C4) are highly expressed in hepatocytes but not in glioma cells, we expect only marginal transformation to MHD in our in vitro proliferation assay [17]. In summary, this suggests that due to the missing inhibitory effects of eslicarbazepine and (R)-MHD, oxcarbazepine itself is most likely responsible for the observed growth inhibition.

### 3.3. Proapoptotic Effect of Oxcarbazepine

**Figure 4:**
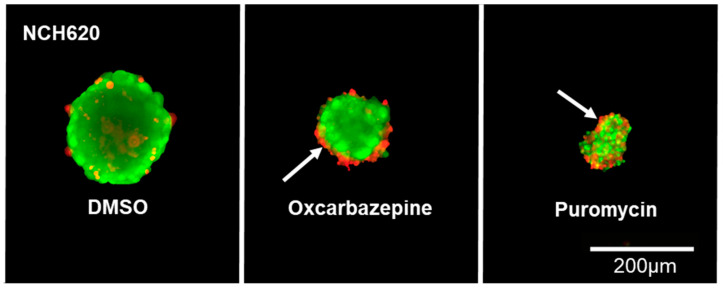
Antiproliferative effect of oxcarbazepine in NCH620. Representative GSC spheroids. After dissociation into a single-cell suspension, oxcarbazepine was added and stained using live/dead staining, showing a reduced size (growth inhibition) upon treatment with oxcarbazepine (30 µM) compared to the DMSO and puromycin (4 µM) controls. Dead cells (red) seem to accumulate at the surface of the treated spheroids, suggesting that oxcarbazepine induces apoptosis and that dead cells are being shuttled outward before being shed (white arrow).

**Figure 5:**
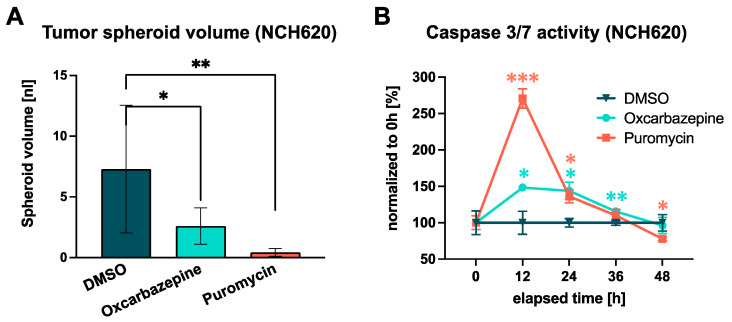
Impact of oxcarbazepine on tumor spheroid growth and the induction of apoptosis in NCH620. (**A**) Comparison of tumor spheroid volumes in a single well of each treatment condition, showing a significant growth inhibition for oxcarbazepine and puromycin. (**B**) Time-resolved induction of apoptosis measured through caspase-3/7 activity. A significant increase in apoptosis after incubation with oxcarbazepine (30 µM) was seen after 12 h. Puromycin served as a positive control (4 µM). * *p* ≤ 0.05; ** *p* ≤ 0.01; *** *p* ≤ 0.001.

Since oxcarbazepine, and not MHD, is able to reduce cell viability, we sought to elucidate its underlying mechanism. The induction of apoptosis under drug treatment was visualized in tumor spheroids using live/dead staining and subsequent fluorescent imaging (Figure 4). The diameter of all spheroids was measured, and the spheroid volume was calculated. The known proapoptotic effect of puromycin was used as a positive control. Indeed, after 72 h, the oxcarbazepine-treated spheroids were 82% smaller than the control spheroids (1.6 nL vs. 8.7 nL; *p* = 0.01), showing that the growth is being inhibited by the drug (Figure 4 and Figure 5A). The accumulation of dead cells (red) at the surface of the treated spheroids compared to the uniformly scattered dead cells in the DMSO control spheroids suggests that the apoptotic cells are being shuttled outward before being shed into the medium (white arrow; Figure 4).

The apoptosis-inducing property of oxcarbazepine was validated by a luminescent caspase-3/7 activity assay with serial measurements over a time course of 48 h. This revealed that the apoptosis induced by oxcarbazepine and puromycin reached its maximum after 12 h (+48.4%; *p* = 0.026; Figure 5B). This effect remained at a higher level for 24 h before decreasing to the level of the DMSO control after 48 h. Taken together, oxcarbazepine exerts a substantial proapoptotic effect on IDH^mut^ GSCs.

## 4. Discussion

This is the first study to conduct a comprehensive repurposing screen of 20 FDA-approved antiepileptic drugs in order to exploit additional antineoplastic effects in IDH^mut^ GSCs. From this library, we found an antiproliferative effect for oxcarbazepine and only moderate and inconsistent effects for perampanel in IDH^mut^ GSCs.

Tumor-associated epilepsy represents a substantial challenge to clinicians, especially in IDH^mut^ glioma patients, as seizures occur more frequently in this patient population than in other glioma entities [18]. While numerous anti-seizure medications exist, less attention has been given to the additional effects that these drugs may have on tumor progression [19]. Particularly in IDH^mut^ glioma, preclinical data are difficult to obtain as tumor models are still sparse compared to IDH^wt^ glioma models. This is, in part, due to difficulties in establishing valid cell culture models where the natural genetic background of the IDH mutation is maintained, as compared to most tumor models where the mutation is synthetically introduced [20,21]. However, in the past, we have successfully used six patient-derived IDH^mut^ GSCs to identify several FDA-approved drugs [13]. While the tested drugs were known antineoplastics, the present study aims to repurpose antiepileptics as anti-cancer drugs.

The tested drug library comprises 20 FDA-approved AEDs selected by literature reviews that are currently or have been previously used in clinical practice. The decision of which AEDs to use is based on evidence generated mainly for non-tumor-related epilepsy, and only low-level data exist for treating TAE (Table 2) [22,23]. Nonetheless, levetiracetam, phenytoin, and pregabalin are considered first-line treatments in TAE. Carbamazepine, lacosamide, oxcarbazepine, topiramate, perampanel, and valproate are being administered if first-line monotherapy fails and can be given instead or as add-on therapy [23,24,25]. The remaining AEDs are less commonly used in TAE. The modes of action of how these drugs are thought to exert their anti-seizure effects are diverse and, in part, overlap (Figure 1; Table 2). Most AEDs (phenytoin, carbamazepine, eslicarbazepine, rufinamide, lacosamide, oxcarbazepine, lamotrigine, zonisamide, ethosuximide, felbamate, topiramate, gabapentin, and pregabalin) modify the electrolyte flux (Na^+^, Ca^2+^, K^+^) through the neuronal cell membrane, thus reducing its excitability and stabilizing the electric potential of the neuronal cell membrane. Other AEDs (tiagabine, vigabatrin, and valproate) act by boosting the inhibitory neurotransmission mediated by gamma-aminobutyric acid (GABA), either by increasing its availability or by decreasing its breakdown [26]. Besides that, additional beneficial pharmacological effects might be induced. Among the AEDs that have an antiproliferative activity, valproic acid is the best studied one [27]. It is a good example of how a drug may be repurposed, as its antineoplastic effect is attributable to its HDAC inhibition and not to its GABA-mediating effect [28]. This could be shown in numerous preclinical studies but translates into somewhat contradictory clinical results [28,29,30,31,32,33,34,35,36]. However, in this study, valproic acid did not affect the proliferation of IDH^mut^ GSCs. Primidone and stiripentol are considered positive allosteric modulators of the GABA_A_ receptor, thus enhancing its inhibitory effect upon stimulation with GABA [37]. Perampanel selectively blocks the excitatory AMPA receptor [38]. Levetiracetam binds to the presynaptic vesicle protein SV2A, which then limits the release of the excitatory neurotransmitter glutamate [39].

In the initial screen, oxcarbazepine and perampanel were identified to exert growth inhibition in four out of the six and one out of the six GSCs, respectively. The subsequent validation experiments showed that oxcarbazepine had a mean IC_50_ value of 44.7 µM in five out of the six GSCs (17.4–98.6 µM). Perampanel showed a dose-dependent response but did not reach the IC_50_ in the range of the concentrations tested. The fluorescent visualization of the apoptotic cells and the caspase-3/7 activity measurements revealed that oxcarbazepine induces growth inhibition through a proapoptotic effect. This study discovered oxcarbazepine as a promising antineoplastic antiepileptic drug for the treatment of IDH^mut^ glioma, which should be taken into further preclinical and clinical testing.

Physiologically, oxcarbazepine is extensively metabolized in the cytosol of hepatocytes to MHD, which exerts the antiepileptic effect by blocking the voltage-gated sodium channels [44]. It exists as two enantiomers, which are (S)-MHD (eslicarbazepine) and (R)-MHD [45]. Several aldo-keto reductases are responsible for the metabolization of oxcarbazepine (AKR1C1, AKR1C2, AKR1C3, and AKR1C4), which have not been found to be expressed in the brain or in gliomas [17]. This also indicates that no substantial transformation of oxcarbazepine to MHD happened during the assessed time frame of our in vitro analysis. The antiepileptic therapeutic range of MHD in patients was found to be between 13 and 133 µM (mean 57 µM) [46]. Interestingly, neither enantiomer showed any effect in our analyses, suggesting that the unmetabolized oxcarbazepine or yet another metabolite must induce the observed cell growth inhibition that is not attributable to the blockade of membrane ion channels. This is in line with a previous report analyzing the effect of oxcarbazepine and both MHD enantiomers given at the equivalent dose in U87MG and T98G glioblastoma (GBM) cell lines, both IDH wildtypes [47]. When considering clinical applications, the metabolization of oxcarbazepine into MHD must, therefore, be taken into account. Still, in a study analyzing the maximum serum concentrations (c_max_) of oxcarbazepine and MHD following a single oral administration of oxcarbazepine (30 mg/kg), oxcarbazepine was able to reach 24 µM, which may indicate that an effective antineoplastic dose could be reached [48]. Another way of overcoming liver metabolism may be by designing modifications of oxcarbazepine in such a way that they are not significantly metabolized by the liver.

The few clinical studies analyzing the possible anticancer effects of oxcarbazepine were not specifically designed to investigate its antineoplastic activity. A retrospective, nation-wide Norwegian registry study analyzed the effects of several AEDs, including oxcarbazepine, on GBM patients. Although the authors did not find a significant effect on overall survival, it is questionable if this is also the case for IDH^mut^ gliomas since the study population was not stratified by IDH mutation status [33]. Furthermore, the combinatorial treatment of oxcarbazepine with other antineoplastic agents has not yet been the focus of any studies, and it might reveal synergistic effects that add value to existing therapies. Assessing the anti-tumor effect of oxcarbazepine in an IDH^mut^ glioma mouse model would be necessary to close the gap between our in vitro findings and the scarce clinical evidence.

The reasoning for the use of antiepileptics in glioma therapies has been further boosted by preclinical studies analyzing the interplay between neurons and glioma cells. Venkataramani et al. and Venkatesh et al. suggest that neuronal hyperexcitation via glutamate release stimulates the bona fide glutamatergic synapses on glioma cells, which, in turn, leads to tumor progression [8,9]. Antagonizing the AMPA glutamate receptor with perampanel in xenografted mice decreased the proliferation of GBM cells, as determined by in vivo imaging [8]. In another study, perampanel inhibited tumor growth in primary GBM cell cultures but failed to mitigate tumor growth or promote a survival benefit in C6 glioma rats [49,50]. Likewise, our AED screen in IDH^mut^ GSCs showed that perampanel inhibited growth in only one specific GSC (NCH620) at a concentration of 10 µM. In subsequent experiments, a dose–response relationship could be seen, although the IC_50_ was not reached in the range of the concentrations tested. Whether this decreased sensitivity compared to IDH^wt^ GSCs is due to the underlying biological differences between IDH^wt^ and IDH^mut^ gliomas remains to be elucidated. Whether oxcarbazepine also influences the glioneuroal network should be the subject of upcoming studies.

When treating IDH^mut^ glioma patients, one will inevitably be confronted with the treatment of tumor-associated epilepsy. The choice of antiepileptic medication has so far been guided by the effectiveness of its anti-seizure capability. This study provides preclinical reasoning for the use of oxcarbazepine beyond the scope of its antiepileptic purpose. Further analyses will be required to reveal its antineoplastic mode of action, which may help to combine it with already established chemotherapeutics.

## 5. Conclusions

This repurposing drug screen with 20 antiepileptic drugs identifies oxcarbazepine as an antiproliferative drug in IDH mutant glioma stem cells. The antiproliferative effect is mediated through its proapoptotic property. These results could serve as a basis for integrating the antiepileptic drug into established chemotherapies for patients suffering from epileptogenic IDH mutant gliomas.

## Figures and Tables

**Table 1:** Characteristics of parental glioma patients and tumor-derived glioma stem-like cells.

GSCs	Diagnosis	IDH Mutation	Gender	TAE
NCH612	rOligo WHO grade 3	IDH mutated ^#^	m	yes
NCH1681	rAstro WHO grade 3	IDH1 (R132H) *	f	yes
NCH551b	rAstro WHO grade 4	IDH1 (R132H) *	m	yes
NCH620	rAstro WHO grade 4	IDH1 (R132H) *	f	yes
NCH645	rAstro WHO grade 4	IDH1 (R132H) *	m	no
NCH3763	rAstro WHO grade 4	IDH1 (R132H) *	m	yes

GSCs = glioma stem-like cells; rAstro/Oligo= recurrent astrocytoma/oligodendroglioma; m = male; f = female; TAE = tumor-associated epilepsy; ^#^ determined by IHC. * determined by sequencing.

**Table 2:** Mode of action, usage, and therapeutic reference range of antiepileptic drugs [23,40,41,42,43].

	Mode of Action										Usage		Therapeutic Reference Range [41,42,43]	
	Na^+^ Channel	Ca^2+^ Channel	GABAa Rec. Modidifier	Modul. of GABA Disposition	Ca^2+^ Dependent K^+^ Channel	CA Inhibitor	AMPA Receptor	Kainate Receptor	SV2A Vesicle Binding	HDAC Inhibitor	Efficacy in Glioma [23]	Adults with Partial Onset Seizures [40]	µg/mL	µM
Phenytoin	+++										***	Level A	10–20	40–80
Carbamazepine	+++										**	Level A	4–11	15–45
Eslicarbazepine	++												3–26	12–100
Rufinamide	++												4–31	15–130
Lacosamide	++									+	**		3–10	10–40
Oxcarbazepine ^a^	++	++								+	**	Level C	3–36	12–140
Lamotrigine	++	+++										Level C	3–13	10–50
Zonisamide	+	+				+						Level A	10–38	45–180
Ethosuximide	+	+			+								39–99	280–700
Felbamate	+	+	+										30–60	125–250
Topiramate	+	+	+			+		++			**	Level C	2–10	6–30
Gabapentin		+										Level C	3–21	20–120
Pregabalin		+									***		2–6	10–35
Tiagabine				+++									0.020–0.100	0.05–0.25
Vigabatrin				+++								Level C	0.8–36	6–278
Valproate	+	+	+	+++						+++	**	Level B	43–101	300–700
Primidone ^b^			+++									Level D	12–30	50–130
Stiripentol			+++										4–22	15–95
Perampanel							+++				**		0.1–1	0.25–2.85
Levetiracetam		+	+				+		+++		***	Level A	5–41	30–240

Abbreviations: rec. = receptor; GABA = gamma-aminobutyric acid; HDAC = histone deacetylase; AMPA = α-amino-3-hydroxy-5-methyl-4-isoxazolpropionic acid; SV2A = synaptic vesicle glycoprotein 2A; ^a^ reference range applies to the active metabolite monohydroxy derivative (MHD); ^b^ reference range applies to the active metabolite phenobarbital; +++ certain; ++ likely; + possible; *** first-line monotherapy; ** second-line or add-on therapy.

## Data Availability

The data presented in this study are available on request from the corresponding author. The data are not publicly available due to privacy reasons.

## Supplementary Materials

The following supporting information can be downloaded at https://www.mdpi.com/article/10.3390/cells12081200/s1, Figure S1: Dose–response curves of (R) monohydroxy derivative (MHD) in GSC lines in which oxcarbazepine has shown an effect. (R)-MHD is one of the two metabolites of oxcarbazepine thought to exert an antiepileptic effect. In all of the tested glioma stem cell lines, (R)-MHD was not able to induce growth inhibition, suggesting that oxcarbazepine itself promotes the antineoplastic effect.
