# Supplementary material for: The Antiepileptic Drug Oxcarbazepine Inhibits the Growth of Patient-Derived Isocitrate Dehydrogenase Mutant Glioma Stem-like Cells
Source: Cells. 2023 Apr 20;12(8):1200. doi: 10.3390/cells12081200 (PMC10136933; doi:10.3390/cells12081200)
Supplement: Supplementary file 1 [file cells-12-01200-s001.zip › cells-2286320-supplementary.pdf]

Supplementary Material

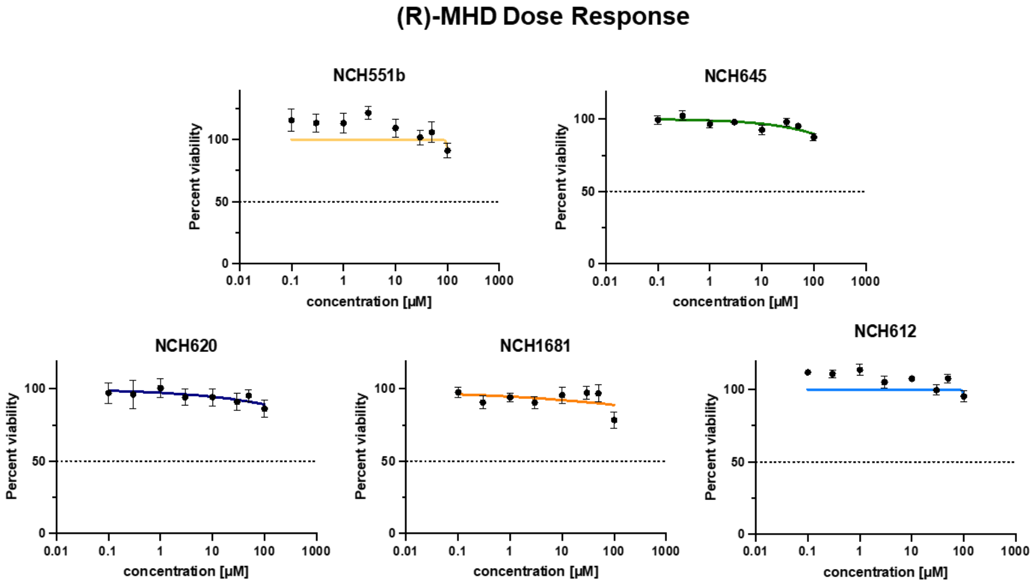

Supplementary Figure S1: Dose–response curves of (R) monohydroxy derivative (MHD) in GSC lines in which oxcarbazepine has shown an effect. (R)-MHD is one of the two metabolites of oxcarbazepine thought to exert an antiepileptic effect. In all of the tested glioma stem cell lines, (R)-MHD was not able to induce growth inhibition, suggesting that oxcarbazepine itself promotes the antineoplastic effect.S
